# Alopecia Areata Increases the Risk of Stroke: a 3-year Follow-Up Study

**DOI:** 10.1038/srep11718

**Published:** 2015-06-26

**Authors:** Jiunn-Horng Kang, Herng-Ching Lin, Senyeong Kao, Ming-Chieh Tsai, Shiu-Dong Chung

**Affiliations:** 1 Department of Physical Medicine and Rehabilitation, Taipei Medical University Hospital, Taipei, Taiwan; 2 Department of Physical Medicine and Rehabilitation, School of Medicine, College of Medicine, Taipei Medical University, Taipei, Taiwan; 3 Sleep Research Center, Taipei Medical University Hospital, Taipei, Taiwan; 4 School of Public Health, National Defense Medical Center, Taipei, Taiwan; 5 Division of Gastroenterology, Department of Internal Medicine, Cathay General Hospital, Taipei, Taiwan; 6 Division of Urology, Department of Surgery, Far Eastern Memorial Hospital, New Taipei City, Taiwan; 7 School of Medicine, Fu-Jen Catholic University, New Taipei City, Taiwan

## Abstract

The risk for stroke in alopecia areata (AA) patients is still unknown. This study aimed to investigate the risk for subsequent risk of a stroke in AA patients in a large-scale retrospective cohort study. We identified 3231 patients with AA included in the study group from 2004 to 2011 in the “Longitudinal Health Insurance Database 2000” in Taiwan. We randomly selected 16,155 matched patients as the comparison group. We individually tracked each patient for a 3-year period to identify patients who had received a diagnosis of stroke during the follow-up period. We found that incidence rates of stroke during the 3-year follow-up periods were 5.44 (95% confidence interval (CI) = 4.03 ~ 7.20) and 2.75 (95% CI = 2.30 ~ 3.27) per 1000 person-years for patients with and those without AA, respectively. Cox proportional hazard regressions showed that the adjusted hazard ratio for stroke for those patients with AA was 1.61 (95% CI = 1.13 ~ 2.30) within the follow-up period compared to the controls. We concluded that patients with AA were associated with a higher risk of stroke in the 3-year follow-up period.

Alopecia areata (AA) refers to the loss of hair over regional areas or diffusely over the entire body. AA is usually diagnosed based on specific clinical features. Although the cause of AA is still not fully understood, autoimmune responses that attack hair follicles and suppress hair growth mediated by T cells are considered to be an important pathomechanism of AA^1^,^2^. AA is not generally considered to be a life-threatening disease; nevertheless, AA is a clinically heterogeneous disease with wide variations in presentation and severity^3^,^4^. The response to treatments in the severe form of AA can be poor^5^. In addition, patients with AA may suffer from significant changes in their body image, and the disease is associated with psychological consequences such as anxiety, depression, and social phobias^6^.

Stroke is a common neurological disease and is associated with significant socioeconomic and medical burdens worldwide. In addition to traditional cardiovascular risk factors, several recent reports demonstrated that many other conditions such as depression, inflammation, and infection may play a role in the pathogenesis and risk of a stroke^7^,^8^,^9^,^10^. Systemic autoimmune diseases such as rheumatoid arthritis, systemic lupus erythematosus, and ankylosing spondylitis have been reported to accelerate arthrosclerosis and to be associated with higher risks of cardiovascular diseases (CVDs) including stroke^11^. Aggressive management of inflammation in such patients may help reduce the risk for CVDs and stroke.

To the best of our knowledge, there are still no data on the association between AA and stroke. There are some biological linkages to underlying diseases, pathogeneses, and consequences between AA and stroke noted in the previous literature. Therefore, we hypothesized that patients with AA may be associated with a subsequently increased risk for stroke occurrence. In the present study, we explored the temporal association between AA and stroke with a large-scale population-based retrospective cohort study in Taiwan. These epidemiological data can provide fundamental evidence regarding the association between AA and stroke.

## Methods

### Database

This retrospective cohort study used data retrieved from the “Longitudinal Health Insurance Database 2000” (LHID2000), which is derived from the Taiwan National Health Insurance (NHI) program and released to the public by the Taiwan National Health Research Institute. The LHID2000 contains registration files and claims data of 1,000,000 individuals randomly sampled from the 2000 Registry for Beneficiaries of the NHI program. The LHID2000 provides an exclusive opportunity for researchers to explore the relationship between AA and the subsequent risk of stroke.

This study was exempt from full review by the Institutional Review Board (IRB) of the National Defense Medical Center because the LHID2000 consists of de-identified secondary data released to the public for research purposes. The methods were carried out in accordance with the approved guidelines.

### Study Sample

This follow-up study featured a study group and a comparison group. We first identified a study group by selecting those patients who had received a first-time diagnosis of AA (ICD-9-CM code 704.01, including typical AA and rarely observed AA incognita) during ambulatory care visits between January 2004 and December 2011 (*n* = 4065). We excluded all patients under the age of 18 years in order to limit the study sample to the adult population (*n* = 578). We further assigned the date of their first diagnosis of AA as the index date. Finally, we excluded patients who had a history of any type of stroke (ICD-9-CM code 430 ~ 438) prior to the index date (*n* = 256). As a result, 3231 patients with AA were included in the study group.

For the comparison group, we likewise selected patients from remaining enrollees in the registry of beneficiaries of the LHID2000. We first excluded all enrollees aged less than 18 years and those with a history of AA. Thereafter, we randomly selected 16,155 patients (five for every patient with AA) to match the study group in terms of sex, age group (18 ~ 39, 40 ~ 49, 50 ~ 59, 60 ~ 69, and >69 years), and the year of the index date. For the comparison group, their first medical service utilization occurring during the index year was designated as their index date. In addition, we assured that all selected comparison patients had no history of stroke prior to their index date.

Ultimately, 19,386 sampled patients were included in this study. We individually tracked each patient for a 3-year period starting from their index date in order to identify patients who had received a diagnosis of stroke during this follow-up period. Furthermore, we censored patients who died from non-stroke causes during the 3-year follow-up period; 821 patients died from non-stroke causes, comprising 142 from the study group (4.4% of the study group) and 679 from the comparison group (4.2% of the comparison group).

### Statistical Analysis

SAS System for Windows, vers. 8.2 (SAS Institute, Cary, NC) was used for the statistical analyses in this study. We used Pearson ^χ2^ tests to examine differences in sociodemographic characteristics (monthly income and geographic location) as well as selected comorbidities between patients with and those without AA. The comorbidities selected for adjustment included hypertension, diabetes, coronary heart disease (CHD), heart failure, atrial fibrillation, and hyperlipidemia. We selected these comorbidities because they have all been reported to be potential risk factors for stroke. In addition, we only included these comorbidities if they occurred in an inpatient setting or in two or more ambulatory care claims coded within 1 year before or after the index date.

We also used log-rank test to explore the difference in 3-year stroke-free survival rates between the study and comparison groups. We used Cox proportional hazard regressions (stratified by sex, age group, and the year of the index date) to examine the relationship between AA and subsequent stroke during the 3-year follow-up period. Hazard ratios (HRs) with 95% confidence intervals (CIs) were computed to present the risk of stroke using a significance level of 0.05.

## Results

Table 1 presents the distributions of sociodemographic characteristics and comorbidities for the sampled patients according to the presence/absence of AA. The mean age of sampled patients was 36.2 years, with a standard deviation of 12.2 years, and they were 36.1 and 36.2 years for patients with and those without AA. Our analysis showed that after matching for sex, age group, and the index year, patients with AA were more likely to have a monthly income of > New Taiwan (NT)$25,000 (*p* < 0.001) and to reside in northern Taiwan (*p* = 0.026) than those without AA. In addition, there was a significant difference in the distribution of the prevalence of CHD (*p* < 0.001) and hyperlipidemia (*p* < 0.001) between patients with and those without AA.

Table 2 shows the incidence of stroke during the 1-year follow-up period according to the presence or absence of ischemic bowel disease. Incidence rates of stroke during the 3-year follow-up period were 3.17 (95% CI = 2.73 ~ 3.67), 5.44 (95% CI = 4.03 ~ 7.20), and 2.75 (95% CI = 2.30–3.27) per 1000 person-years for all sampled patients, and those with and those without AA, respectively. The log-rank test suggested that patients with AA had a significantly lower 3-year stroke-free survival rate than patients without (*p* = 0.004).

Furthermore, Table 2 shows the HR and its corresponding 95% CI for stroke between patients with and those without AA. After adjusting for urbanization level, geographic region, monthly income, CHD, and hyperlipidemia, the HR for stroke for those patients with AA was 1.61 (95% CI = 1.13 ~ 2.30) within the 3-year follow-up period compared to patients without AA.

Table 3 shows the HRs for stroke by stroke type. It reveals that patients with AA consistently had a higher hazard of stroke within the 3-year follow-up period than patients without AA regardless of stroke type. Adjusted HRs for hemorrhage stroke, ischemic stroke, and “unspecified” stroke were 2.18 (95% CI = 1.01 ~ 4.84), 1.58 (95% CI = 1.00 ~ 2.45), and 2.27 (95% CI = 1.19 ~ 4.31), respectively, compared to patients without AA.

## Discussion

Our study showed that patients with AA were associated with a higher risk (adjusted HR of 1.61) of stroke occurrence in the 3-year follow-up period compared to controls from a large population-based database in Taiwan. To the best of our knowledge, this is the first report to indicate an association between AA and stroke. This epidemiological finding supports our hypothesis that patients with AA may have a higher risk for a subsequent stroke. Although the mechanism of the association is currently still unknown, paying closer attention to detecting and monitoring underlying cardiovascular risks in patients with AA may be needed in clinical settings. Further study is advised to explore the underlying pathomechanism of the association of AA and stroke to develop specific management strategies to reduce the stroke risk in AA patients.

Currently, there is no evidence or report regarding a direct cause and effect linkage between AA and stroke. The cause of this association could be multifactorial and complex. Several levels of biological linkages between AA and stroke can be hypothesized. Shared underlying risk factors should be noted in explaining the association between AA and stroke from the literature. Atopic diseases and systemic autoimmune diseases including systemic lupus erythematosus, rheumatoid arthritis, inflammatory bowel disease, and psoriasis were shown to be more prevalent in AA patients^12^. The chronic inflammatory status in these systemic diseases is considered to be a promoting and accelerating factor of arthrosclerosis and stroke^11^. In addition to inflammatory disorders, a higher prevalence of thyroid disorders and dysfunction was also reported in patients with AA^1^,^12^. Thyroiditis, hypothyroidism, and hyperthyroidism can alter the coagulant status and may increase the risk of stroke^13^,^14^,^15^.

We found that AA patients had higher prevalences of hyperlipidemia and CHDs in the present study. This finding is partially consistent with a recent hospital-based study by Huang *et al.*^12^ These findings imply that cardiovascular risk factors may be more prevalent in AA patients, which may further put these patients at particular risk for CVDs and stroke. It is worth noting that even after we adjusted for these comorbid risks, AA patients still had a higher risk of a stroke during the follow-up period in the present study. Therefore, the increased risk of stroke cannot be explained only by the higher prevalence of these traditional risk factors in AA patients.

Autoimmune responses in hair follicles play an important role in the pathomechanism of AA patients. However, whether the focal autoimmune response in AA patients is severe enough to elicit systemic arthrosclerosis is still unknown. One study showed that levels of serum homocysteine and blood high-sensitivity C-reactive protein, two known independent serum risk factors for stroke^16^,^17^, did not significantly differ between AA patients and controls^18^. Although there is still some controversy, increased oxidative stress in AA patients was reported. Bakry *et al.* found increased oxidative stress and lipid peroxidation in AA patients. Furthermore, the severity of AA was associated with the level of oxidative stress^19^. Yousefi *et al.* found that the level of erythrocyte folate was significantly lower in AA patients than in controls. The level of red blood cell (RBC) folate was also significantly lower in patients with the extensive form and high severity of AA^18^. Therefore, we hypothesized that severe AA patients may have significant pathophysiological changes in their inflammatory and oxidative statuses which may be associated with arthrosclerosis. Further study is needed to evaluate cardiovascular risks according to the severity and pattern of AA patients.

Consequent psychological stress and illnesses associated with AA are other potential factors linking AA to stroke occurrence. Although it is still equivocal whether stressful life events are associated with triggering AA, patients with AA had higher prevalences of alexithymia, depression, and anxiety compared to the general population^6^,^12^. Detrimental impacts on the quality of life and mental health of AA patients were reported. Mental health problems were suggested to increase CVDs and stroke. A meta-analysis indicated an increased risk of stroke in patients with depression^20^. From a clinical viewpoint, adequate interventions for psychological derangement should be emphasized in AA patients due to its impacts on the quality of life and also its role in linkages to CVDs.

Our study has the advantage of providing sufficient statistical power and lower sampling bias by including a large sample cohort size and nationwide sampling. Nevertheless, results from our study may suffer from some statistical biases. Patients with AA may have increased attention toward other health problems which might have increased the chance of discovering other medical conditions, which is known as ascertainment bias. However, this ascertainment bias might not be a severe problem in discovering stroke occurrence, because a stroke can cause significant neurological symptoms that are generally noted by a patient. There are also several disadvantages from the present study. First, the severity and pattern of AA could not be determined from the registry. Severe forms of AA may be associated with more-pronounced systemic responses which theoretically may be associated with higher risks for stroke occurrence. Second, we did not analyze the effects of treatments of AA on stroke occurrence. There are several treatment strategies for AA. Currently, no data are available on whether the risk for stroke is modified following treatment for AA. Third, some patients with mild AA disease might not have sought medical treatment resulting in their absence from the registry. This condition may have contributed to a mis-estimation of the risk for stroke in the present study. Fourth, family history of AA, dietary habits, physical activity, and substance abuse are confounding variables which could not be determined in the present study. Fifth, miscoding must be considered when analyzing a medical registry database.

In conclusion, patients with AA were associated with an increased risk for a subsequent stroke in the 3-year follow-up period in Taiwan. Further study is advised to confirm our findings and explore the underlying pathomechanism.

## Additional Information

**How to cite this article**: Kang, J.-H. *et al.* Alopecia Areata Increases the Risk of Stroke: a 3-year Follow-Up Study. *Sci. Rep.* **5**, 11718; doi: 10.1038/srep11718 (2015).

## Figures and Tables

**Table 1:** Demographic characteristics of subjects with alopecia areata (AA) and comparison subjects (n = 19,386).

**Variable**	**Subjects with AA,** ***N***** = 3231**		**Comparison subjects** ***N***** = 16,155**		***p*** **value**
	**Total no.**	**Column %**	**Total no.**	**Column %**	
Sex					>0.999
Male	1588	49.2	7940	49.2	
Female	1643	50.8	8215	50.8	
Age group (years)					>0.999
18 ~ 39	2113	65.4	10,565	65.4	
40 ~ 49	637	19.7	3185	19.7	
50 ~ 59	318	9.8	1590	9.8	
60 ~ 69	117	3.6	585	3.6	
>69	46	1.4	230	1.4	
Geographic region					0.026
Northern	1744	54.0	8334	51.6	
Central	707	21.9	3586	22.2	
Southern	719	22.3	3960	24.5	
Eastern	61	1.8	275	1.7	
Monthly income (New Taiwan (NT)$)					<0.001
NT$0 ~ 15,840	1162	36.0	6625	41.0	
NT$15,841 ~ 25,000	991	30.7	5001	31.0	
≥NT$25,001	1078	33.3	4529	28.0	
Hyperlipidemia	642	19.9	2579	16.0	<0.001
Hypertension	560	17.3	2635	16.3	0.153
Diabetes	317	9.8	1456	9.0	0.151
Coronary heart disease	285	8.8	1015	6.3	<0.001
Atrial fibrillation	23	0.7	75	0.5	0.070
Heart failure	41	1.3	185	1.2	0.550

In 2012, the average exchange rate was US$1.00 ≈NT$30.

**Table 2:** Crude and adjusted hazard ratios (HRs) for stroke during the three-year follow-up period for subjects with alopecia areata (AA) and comparison subjects (n = 19,386).

**Stroke occurrence**	**Total sample**	**Subjects with AA** ***N***** = 3231**	**Comparison subjects** ***N***** = 16,155**
Three-year follow-up period			
Incidence rate per 1,000 person-years (95% CI)	3.17 (2.73 ~ 3.67)	5.44 (4.03 ~ 7.20)	2.75 (2.30 ~ 3.27)
Crude HR (95% CI)	—	1.66 ^b^(1.17 ~ 2.35)	1.00
Adjusted ^a^ HR (95% CI)	—	1.61^b^(1.13 ~ 2.30)	1.00

Notes: ^a^Stratified Cox proportional hazard regressions (stratified by sex and age) were performed to adjust for monthly income, geographic region, hyperlipidemia, and coronary heart disease; ^b^*p* < 0.01. CI, confidence interval.

**Table 3:** Crude and covariate-adjusted hazard ratios (HRs) for different types of stroke among sampled patients during the 3-year follow-up period starting from the index date.

**Stroke occurrence**	**Total sample**	**Subjects with alopecia areata**	**Comparison subjects**
Hemorrhagic stroke			
Incidence rate per 1000 person-years (95% CI)	0.51 (0.34 ~ 0.74)	1.01 (0.46 ~ 1.90)	0.42 (0.26 ~ 0.65)
Crude HR (95% CI)	–	2.26^a^ (1.03 ~ 4.97)	1.00
Adjusted HR (95% CI)	–	2.18^a^ (1.01 ~ 4.84)	1.00
Ischemic stroke			
Incidence rate per 1000 person-years (95% CI)	1.88 (1.54 ~ 2.27)	2.89 (1.89 ~ 4.23)	1.69 (1.34 ~ 2.11)
Crude HR (95% CI)	–	1.63^a^ (1.05 ~ 2.55)	1.00
Adjusted HR (95% CI)	–	1.58^a^ (1.00 ~ 2.45)	1.00
Unspecified			
Incidence rate per 1000 person-years (95% CI)	0.78 (0.57 ~ 1.05)	1.56 (0.85 ~ 2.61)	0.63 (0.43 ~ 0.91)
Crude HR (95% CI)	–	2.35^b^ (1.24 ~ 4.43)	1.00
Adjusted HR (95% CI)	–	2.27^a^ (1.19 ~ 4.31)	1.00

Notes: CI, confidence interval; the HR was calculated using the Cox proportional hazard regression, and was stratified by sex, age group, and the year of the index date; Adjusted for monthly income, geographic region, hyperlipidemia, and coronary heart disease; ^a^*p* < 0.05; ^b^*p* < 0.01.
